# Specialization-generalization trade-off in a *Bradyrhizobium* symbiosis with wild legume hosts

**DOI:** 10.1186/1472-6785-14-8

**Published:** 2014-03-19

**Authors:** Martine Ehinger, Toni J Mohr, Juliana B Starcevich, Joel L Sachs, Stephanie S Porter, Ellen L Simms

**Affiliations:** 1 Department of Integrative Biology, University of California, Berkeley, CA, USA; 2 Department of Biology, University of California, Riverside, CA, USA; 3 Institute of Integrative Genomic Biology, University of California, Riverside, CA, USA

**Keywords:** Mutualism, Symbiosis, Specialization, Coevolution, *Lupinus bicolor* (dove lupine), *Acmispon strigosus* (strigose trefoil), *Lotus*, *Alpha-proteobacteria*, Legumes, Rhizobia

## Abstract

**Background:**

Specialized interactions help structure communities, but persistence of specialized organisms is puzzling because a generalist can occupy more environments and partake in more beneficial interactions. The “Jack-of-all-trades is a master of none” hypothesis asserts that specialists persist because the fitness of a generalist utilizing a particular habitat is lower than that of a specialist adapted to that habitat. Yet, there are many reasons to expect that mutualists will generalize on partners.

Plant-soil feedbacks help to structure plant and microbial communities, but how frequently are soil-based symbiotic mutualistic interactions sufficiently specialized to influence species distributions and community composition? To address this question, we quantified realized partner richness and phylogenetic breadth of four wild-grown native legumes (*Lupinus bicolor*, *L. arboreus, Acmispon strigosus* and *A. heermannii*) and performed inoculation trials to test the ability of two hosts (*L. bicolor* and *A. strigosus*) to nodulate (fundamental partner richness), benefit from (response specificity), and provide benefit to (effect specificity) 31 *Bradyrhizobium* genotypes.

**Results:**

In the wild, each *Lupinus* species hosted a broader genetic range of *Bradyrhizobium* than did either *Acmispon* species, suggesting that *Acmispon* species are more specialized. In the greenhouse, however, *L. bicolor* and *A. strigosus* did not differ in fundamental association specificity: all inoculated genotypes nodulated both hosts. Nevertheless, *A. strigosus* exhibited more specificity, i.e., greater variation in its response to, and effect on, *Bradyrhizobium* genotypes. *Lupinus bicolor* benefited from a broader range of genotypes but averaged less benefit from each. Both hosts obtained more fitness benefit from symbionts isolated from conspecific hosts; those symbionts in turn gained greater fitness benefit from hosts of the same species from which they were isolated.

**Conclusions:**

This study affirmed two important tenets of evolutionary theory. First, as predicted by the Jack-of-all-trades is a master of none hypothesis, specialist *A. strigosus* obtained greater benefit from its beneficial symbionts than did generalist *L. bicolor*. Second, as predicted by coevolutionary theory, each test species performed better with partner genotypes isolated from conspecifics. Finally, positive fitness feedback between the tested hosts and symbionts suggests that positive plant-soil feedback could contribute to their patchy distributions in this system.

## Background

Specialized biotic interactions contribute to processes that structure communities [1] and adapt populations to local partners and environments [2,3]. But, why do specialists exist if a generalist organism can occupy more environments and partake in more beneficial interactions [4,5]? The “Jack-of-all-trades is a master of none” hypothesis asserts that specialists persist in a heterogeneous environment because the fitness of a generalist utilizing a particular habitat is lower than that of a specialist adapted to that habitat [5-7]. This hypothesis is based on the assumptions that adapting to new habitats involves fitness costs and that traits adaptive in one subset of environments are negatively genetically correlated with traits adaptive in other environments [4,8-11], leading to local adaptation [2,3]. When the habitat is a host or interaction partner, this trade-off could lead to co-adaptation [12].

Fitness feedbacks between plants and specialized soil-based microbes significantly affect plant productivity, community composition, and the distribution and abundance of plants [13-21]. Strong plant-soil feedbacks are often negative; due to antagonistic interactions between plants and pathogens [13,21-27]. However, many plants engage in mutualistic interactions with soil-borne symbionts [6]. Is there sufficient specialization among plant hosts to soil-borne mutualistic symbionts to impact plant community structure and diversity via positive feedbacks?

Multiple arguments predict that mutualists interacting with partners acquired from the environment are likely to be generalists [28]. Since mutualists have higher fitness when partnered than when not, any partner should be better than no partner. If so, then selection should disfavor specialized mutualists (i.e., those with reduced interaction breadth relative to the breadth of available partners) that resist associating with common partners [29]. Similarly, specialized mutualistic lineages should be vulnerable to perturbations in partner populations [30-36] and the geographic distribution and spread of specialized mutualists could be limited by partner availability [37-44]. Generalized mutualists might also access a wider range of environmental conditions by utilizing partners with differing ecological tolerances [45-48]. Finally, evolutionary convergence of traits among mutualists within an interaction group [49-51], but see [52], could reduce variance in partner benefit and thereby weaken the effectiveness of selection to specialize [53,54].

Specialized mutualists nonetheless exist, e.g. [55-57]. Indeed, meta-analysis of bipartite interactions found that mutualistic webs are more specialized than antagonistic ones [1]. Further, mutualism theory predicts that, when available partners vary in quality, selection favors mechanisms by which mutualists can select partners [58-63], which can lead to specialization. However, if more-beneficial partners are rare or distributed unpredictably, then selection for specialization via partner choice might be weak [45,64,65]. How frequently symbiotic mutualistic interactions are specialized is therefore an open question.

Here, we examined specialization in a group of wild legumes that interact with *Bradyrhizobium* genotypes in coastal sand dunes of Sonoma County, California. *Bradyrhizobium* is a genus of rhizobia that can infect legume roots and fix nitrogen endosymbiotically within root nodules in an interaction that is generally mutualistic [66,67]. Dominant genes that either restrict nodulation with or alter the effectiveness of particular rhizobial genotypes have been described in both natural and managed plant populations [57,68], but the effect of such genes on fitness feedbacks has been little studied. Moreover, little is known about whether legume-rhizobium interactions are sufficiently specialized to contribute to structuring communities via plant-soil feedbacks or coevolution.

We quantified the realized richness and phylogenetic breadth of rhizobial symbionts collected from plants growing *in situ*, and measured the frequency with which each host species associated with each of the identified *Bradyrhizobium* genotypes. Association frequencies quantify the interaction [69] or link [70] strength of each pairwise interaction and were used to calculate the Paired Differences Index PDI [71], for each host. This index summarizes variation in a partner’s link strengths [69,70] with all available partners without making any assumptions about their statistical distribution.

Realized association specificity [72,73] is strongly influenced by the local sampling environment [74]. Therefore, we experimentally paired partners under controlled greenhouse conditions to examine fundamental association frequency [4,75] of two host species. We also used the greenhouse experiment to quantify the fitness effect of each host on the inoculated *Bradyrhizobium* genotypes, which is a measure of the host’s potential impact on the rhizobium population and therefore its functional role within the community [76].

Finally, we used these data to test whether this system supports the “Jack-of-all-trades is a master of none” hypothesis, as well as its underlying assumption that, as frequently interacting partners become co-adapted, they become less adapted to other partners and consequently exchange weaker benefits.

## Results

### Isolate collection and sequence datasets

We isolated *Bradyrhizobium* bacteria from nodules of four native California legumes growing on the Bodega Dunes at Bodega Marine Reserve and Sonoma Coast State Park, Sonoma County. Two species, *Acmispon strigosus* and *Lupinus bicolor*, are fast-growing annuals that are patchily distributed across the Bodega Dunes. In contrast, *A. heermannii* is a decumbent suffrutescent perennial and *L. arboreus* is a large upright perennial shrub, both widely distributed across the dunes.

Two DNA regions (*NifD* and *ITS*) were PCR amplified and sequenced from two to eight isolates from each plant, which yielded 84 sequenced isolates, including 81 *NifD* amplicons and 82 *ITS* amplicons (Genbank accession numbers in Additional file 1: Table S1). Sequences that differed by one nucleotide were considered distinct, resulting in 22 *ITS* genotypes, of which 13 occurred in multiple nodules, and 22 *NifD* genotypes, of which 13 occurred in multiple nodules. There were 39 unique genotype combinations.

### *Realized* in situ *association specificity (realized niche breadth)*

The observed PDIs of the four hosts (*L. arboreus*: 0.264, *A. strigosus*: 0.301, *A. heermannii*: 0.395, *L. bicolor*: 0.433) all differed significantly from the expected joint PDI (0.069 ± 95% confidence limit of 0.002) and from each other, which indicates that each of the four hosts specialized on a subset of the available *NifD* types, but differed in degree of specialization; *L. bicolor* was most specialized.

#### Genetic breadth of symbiotic partners

**Figure 1:**
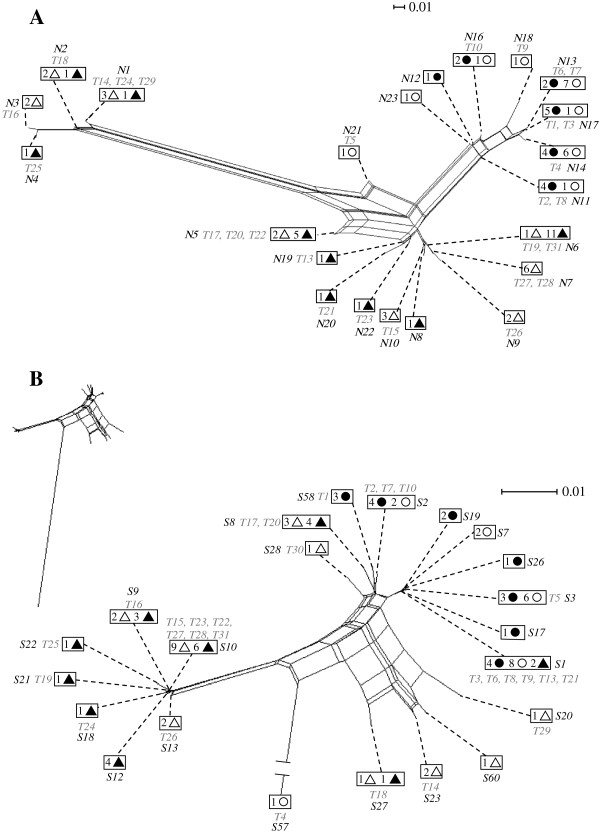
**Genotype networks generated by the neighbor-net algorithm. (A)***NifD* locus, based on the distance calculated by MODELTEST, using a GTR + I model of sequence evolution [121]. **(B)***ITS* region, based on the distance calculated by MODELTEST using a HKY + I + G model of sequence evolution [122]. Dotted lines represent the position of a genotype on the network. Each box represents one genotype, each symbol represents a host species (filled circles represent bacteria harvested from nodules of *A. strigosus*; open circles, *A. heermannii*; filled triangles, *L. bicolor*; open triangles, *L. arboreus*), and numbers indicate the number of strains representing a particular genotype; labels *T1* to *T31* in italics represent the *Bradyrhizobium* genotypes used in the greenhouse experiment (as listed in Additional file 1: Table S1). One genotype, *T30*, appears only in the *ITS* network, because we were unable to sequence its *NifD* locus. Genotypes T11 and T12 do not figure in either network because they were addenda from another study, used for reference, but not part of the original sample.

In both gene networks, *Lupinus* hosts associated with a genetically broader range of bacteria than did *Acmispon* hosts. Isolates from within a host genus tended to cluster together (Figure 1) and exhibited little overlap between host genera. Host genera shared no genotypes in the *NifD* network (Figure 1A). A single common *ITS* genotype (S1) was shared between host genera and one genotype from *Lupinus* (S8) clustered closely to most genotypes from *Acmispon* (Figure 1B).

#### Genetic distance matrices among bacterial communities

Bacterial populations differed significantly between the two host genera but not between species within a genus. For both DNA regions, significantly large Φ_st_ values and corrected between-population mismatch rates occurred between *Bradyrhizobium* populations isolated from different host genera, but not between populations isolated from different host species within a genus (Table 1). However, bacteria isolated from *Lupinus* were more genetically variable than those isolated from *Acmispon*. For both DNA regions, the average pairwise sequence divergences between *Bradyrhizobium* genotypes from *Lupinus* were two- to five-fold greater than they were between genotypes from *Acmispon* (Table 1).

**Table 1:** **Analyses of population structure of *Bradyrhizobium *** **sampled from *in situ *** **nodules**

***(a)**** ITS* region				
	*A. strigosus*	*A. heermannii*	*L. arboreus*	*L. bicolor*
*A. strigosus*	6.6601	0.0422^ns^	0.5814*	0.4405*
*A. heermannii*	0.4506^ns^	13.1696	0.5459*	0.4126*
*L. arboreus*	24.6912*	23.4825*	26.2134	0.0058^ns^
*L. bicolor*	18.1281*	17.9398*	0.0078^ns^	35.922
***(b)**** nifD* locus				
	*A. strigosus*	*A. heermannii*	*L. arboreus*	*L. bicolor*
*A. strigosus*	5.1895	-0.0040^ns^	0.5649*	0.6024*
*A. heermannii*	-0.0217^ns^	7.4971	0.5830*	0.6223*
*L. arboreus*	21.6199*	22.0285*	25.1368	0.0406^ns^
*L. bicolor*	19.3541*	19.6343*	1.0162^ns^	17.0395

Significance, obtained from 1023 permutations: ns, not significant; *, *p* < 0.0001; significant values in boldface.

Sequence divergence (along diagonal), corrected between-population differences (below diagonal), and between-population Φ_st_ (above diagonal).

#### Distribution of bacterial genetic variance

In a two-level hierarchical AMOVA (Table 2), neither *ITS* nor *NifD* sequence explained a significant component of variance between host species within genus. Most genetic variance occurred among plants within species (50.29% for *ITS* and 40.84% for *NifD*) and between host genera (49.14% for *ITS* and 57.92% for *NifD*). Permutations suggest that the variance component due to host genus was not significant (Table 2); even though genera harbored distinct genotypes in both networks (Figure 1) and all inter-genus values of pairwise genetic differences and Φ_st_ were statistically significant for both DNA regions (Table 1). This discrepancy probably arises from a lack of power in the AMOVA, which treats genus as a random effect, even though it is associated with only one degree of freedom.

**Table 2:** **AMOVA of (a) *ITS *** **and (b) *NifD *** **sequences from *Bradyrhizobium *** **from *in situ *** **nodules**

**(a) ***ITS* region					
Sources of variation	df	Sum of squares	variance component	% of variance	P
Between genera	1	440.898	10.5300	49.14	ns
Between species w/in genus	2	26.594	0.1229	0.57	ns
Within species	78	840.667	10.7778	50.29	***
Total	81	1308.159	21.4308		
**(b) ***NifD* locus					
Sources of variation	df	Sum of squares	variance component	% of variance	P
Between genera	1	290.41	10.0195	57.92	ns
Between species w/in genus	2	20.031	0.2149	1.24	ns
Within species	77	416.246	7.06457	40.84	***
Total	80	980.938	17.2989		

Significance: ns, not significant; ***, *p* < 0.001.

Sampled host species: *A. strigosus, A. heermannii, L. bicolor* and *L. arboreus.*

### Fundamental association specificity (fundamental niche breadth)

**Figure 2:**
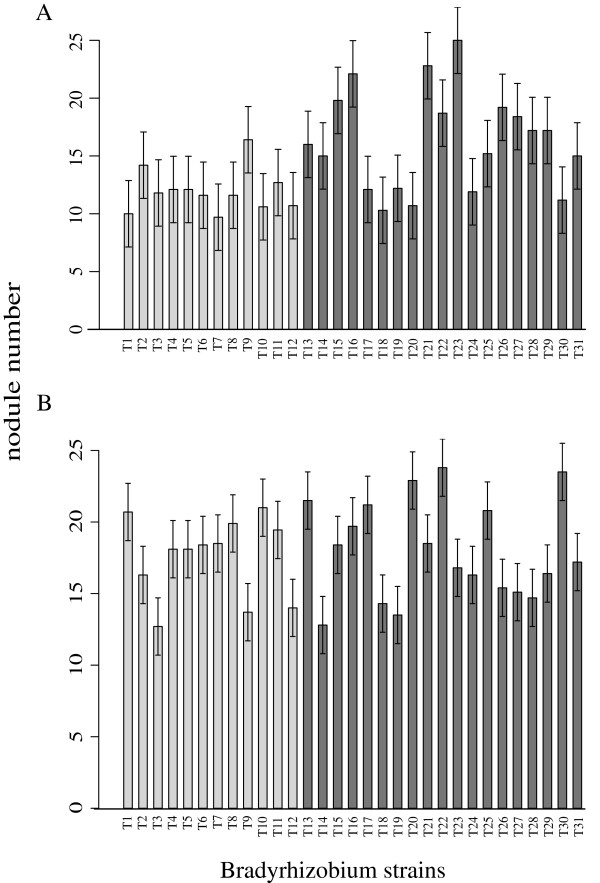
**Average number of nodules produced by each *Bradyrhizobium *genotype on each host species.** Untransformed data. **A)***A. strigosus*. **B)***L. bicolor*. Error bars represent ± 1 standard error. Solid grey bars represent bacteria originally isolated from *A. strigosus* and *A. heermannii* nodules (*A* bacterial group); solid black bars represent bacteria harvested from *L. bicolor* and *L. arboreus* nodules (*L* bacterial group).

**Figure 3:**
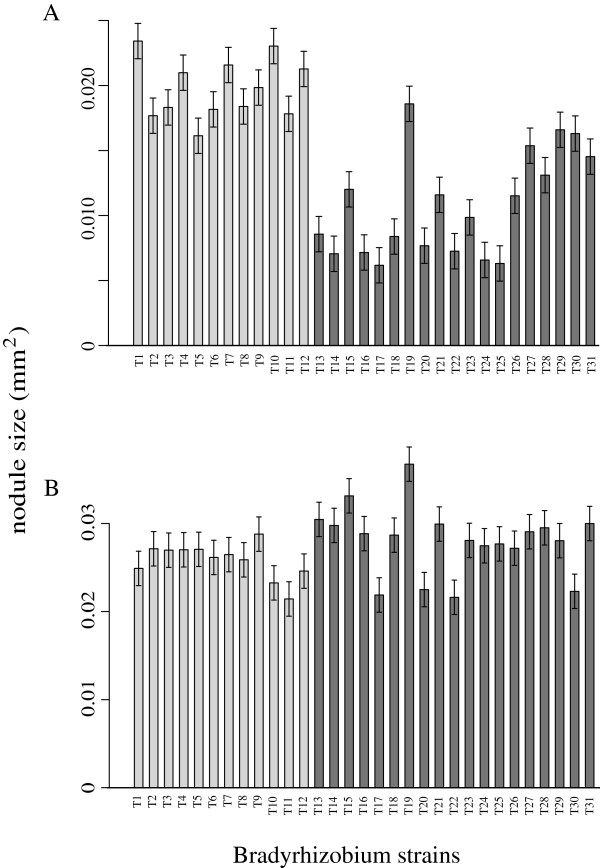
**Average nodule size produced by each *Bradyrhizobium *genotype on each host species.** Untransformed data (cm^2^). **A)***A. strigosus*. **B)***L. bicolor*. Error bars represent ± 1 standard error. Solid grey bars represent bacteria originally isolated from *A. strigosus* and *A. heermannii* nodules (*A* bacterial group); solid black bars represent bacteria harvested from *L. bicolor* and *L. arboreus* nodules (*L* bacterial group).

In the greenhouse inoculation experiment, neither test host exhibited fundamental association specificity; each could nodulate all tested *Bradyrhizobium* genotypes (Figures 2 and 3).

### G × G interaction (Response specificity)

**Figure 4:**
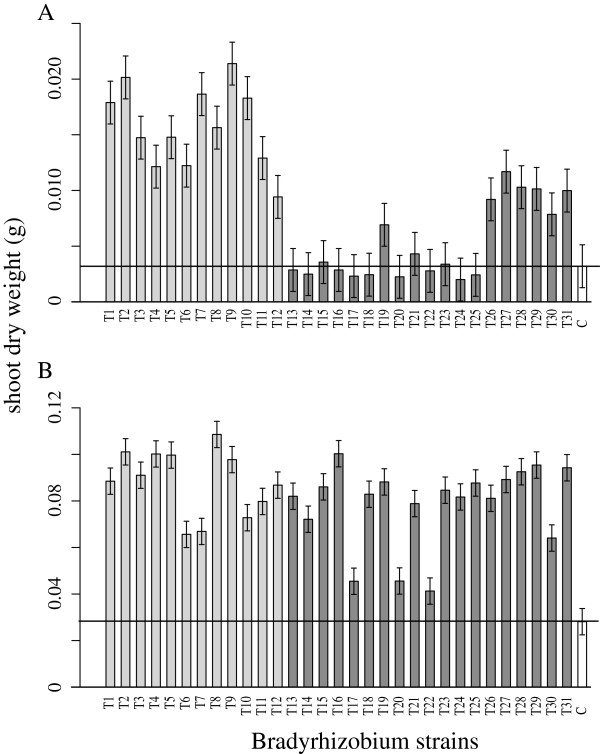
**Mean shoot dry weight of each host species when inoculated by each *Bradyrhizobium*genotype.** Untransformed data (g). **(A) ***A. strigosus*. **(B) ***L. bicolor*. Error bars represent ± 1 standard error. Horizontal reference lines represent mean dry weight of uninoculated control plants. Light grey bars represent bacterial group A, originally isolated from *A. strigosus* and *A. heermannii* nodules; dark grey bars represent bacterial group L, originally isolated from *L. bicolor* and *L. arboreus* nodules; solid white bars represent uninoculated control plants. Genotype order recapitulates that found in Figures 2 and 3.

On average, inoculation increased host shoot dry weight over that of uninoculated control plants. The average increase was 2.8-fold for *A. strigosus* (F_1, 318_ = 64.261, p value < 0.0001) and 2.9-fold for *L. bicolor* (F_1, 314_ = 387.191, p value < 0.0001). However, bacterial genotypes differed significantly in their effects on shoot dry weight (Figure 4, Additional file 2: Table S2 and Additional file 3: Table S3). Notably, several genotypes failed to improve host growth beyond that of uninoculated controls (17 for *A. strigosus* and three for *L. bicolor*, using the Tukey-Kramer honestly significant difference test; 13 for *A. strigosus* and two for *L. bicolor*, using the less conservative Student’s t-test).

The conservative analysis presented above does not test for between-host differences in response specificity, which is typically detected as a significant interaction between test host species and *Bradyrhizobium* genotype G × G interaction [77], in an ANOVA including data from both host species. In a less conservative analysis, host by genotype (G × G) interactions were prominent in the best fitting model (model 16 in Additional file 4: Table S4). A significant G × G interaction of test host with *Bradyrhizobium* groups isolated from different host species (test host by species of origin interaction, *F*_3,25_ = 19.03, *p* > *F* < 0.0001; Additional file 5: Table S5), occurs because variation in response to different *Bradyrhizobium* genotypes was large in *A. strigosus* and almost absent in *L. bicolor* (Additional file 6: Table S6). Further, the G × G interaction due to test host species by *Bradyrhizobium* genotype nested within species of origin (covariance parameter estimate ± 1 S. E. = 0.07235 ± 0.024; Additional file 7: Table S7) accounted for 7.24% ± 2.28% of the variance in plant dry weight. This significant interaction indicates that even within a *Bradyrhizobium* group isolated from the same host species, genotypes differed in the symbiotic benefits they provided to the two different test host species.

As expected from the conservative analysis, test host species differed significantly in dry weight (least square means [logits] of shoot dry weight ± 1 S. E. for *A. strigosus* = -4.80 ± 0.10, *L. bicolor* = -2.40 ± 0.11; t_9_ = -18.36, p < 0.0001) and groups of *Bradyrhizobium* genotypes isolated from different host species differed significantly in average effect on test host shoot dry weight (species of origin effect *F*_3,25_ = 24.56, *p* > *F* < 0.0001; Additional file 8: Table S8). In particular, shoot dry weight produced by genotypes isolated from *L. bicolor* differed from that produced by genotypes isolated from other species (Additional file 6: Table S6 and Additional file 8: Table S8). When averaged across the test hosts, there was no significant variance in shoot dry weight due to inoculation with different *Bradyrhizobium* genotypes isolated from the same host species (strain(sp_orig) covariance parameter estimate ± 1 S. E. = 0.01929 ± 0.020; Additional file 7: Table S7).

**Figure 5:**
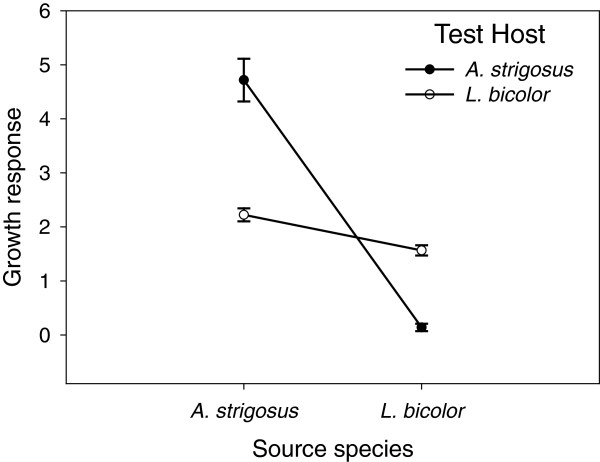
**Mean shoot dry weight response to *Bradyrhizobium *genotypes isolated from conspecific versus allospecific hosts.** Response = fold increase over uninoculated control plants. Closed circles indicate growth of *A. strigosus* test plants; open circles indicate growth of *L. bicolor* test plants. Error bars represent ± 1 standard error.

The fold increase in growth of inoculated plants over uninoculated controls was strongly affected by the interaction of test host with source host (Figure 5; χ^2^_d.f. = 1_ = 25.61, *p* < 0.0001). Further, the planned contrast comparing the fold increase in fitness obtained from *Bradyrhizobium* genotypes isolated from conspecifics versus heterospecifics was highly significant (likelihood ratio χ^2^_d.f. = 2_ = 28.76, *p* < 5 × 10^-7^). Both main effects were statistically significant (test host species χ^2^_d.f. = 1_ = 5.059, *p* < 0.03; host species of origin χ^2^_d.f. = 1_ = 5.059, *p* < 0.03), but the mean growth effect on each host species changed rank between conspecific and heterospecific pairings (χ^2^_d.f. = 1_. = 25.62, *p* = 4 × 10^-7^).

Response specificity, as indicated by the paired difference indices calculated from within-block growth responses stimulated by the different *Bradyrhizobium* genotypes, differed significantly between host species and was more than three-fold greater in *A. strigosus* (mean PDI ± 1 S. E. = 6.6 ± 0.7) than in *L. bicolor* (mean PDI ± 1 S. E. = 1.7 ± 0.1).

### G × G interaction (Effect specificity)

*Bradyrhizobium* fitness was estimated from nodule number, total nodule area, and area of the average nodule. Both nodule number and total nodule area differed significantly among *Bradyrhizobium* genotypes on each host (Additional file 2: Table S2, Figures 2 and 3).

On *A. strigosus*, over 30% of variance in total nodule area was due to *Bradyrhizobium* genotype: genotypes isolated from congeners produced significantly greater total nodule area than did those isolated from lupines. Ten percent of variance in nodule number was due to *Bradyrhizobium* genotype: genotypes isolated from congeners produced slightly fewer nodules than did those isolated from lupines. Seventy percent of variance in average individual nodule area was due to *Bradyrhizobium* genotype: genotypes isolated from congeners produced significantly larger nodules.

On *L. bicolor*, only 15% of variance in total nodule area was explained by *Bradyrhizobium* genotype. Although genotypes isolated from congeners produced significantly greater nodule area, the difference was slight. *Bradyrhizobium* genotype explained almost 15% of variance in nodule number, but there was no significant difference between genotypes isolated from congeners versus *Acmispon* species. Less than 20% of variance in the average individual nodule area on *L. bicolor* was due to *Bradyrhizobium* genotype. Genotypes isolated from congeners produce marginally significantly larger nodules than did those isolated from *Acmispon* hosts.

Paired difference indices (*PDI*) calculated from host effect on *Bradyrhizobium* fitness under non-competitive conditions of single inoculations is presented in Table 3. When measured in terms of nodule number, *A. strigosus* was significantly more specialized than *L. bicolor*, whereas PDI of the two hosts did not differ when calculated in terms of total nodule area.

**Table 3:** **PDI values for two** ***Bradyrhizobium*** **fitness components**

**Fitness component**	***L. bicolor***			***A. strigosus***			**Difference**		
	**Mean PDI**	**LCL**	**UCL**	**Mean PDI**	**LCL**	**UCL**	**Diff PDI**	**LCL**	**UCL**
Nodule area*	0.217	0.189	0.246	0.228	0.186	0.271	0.102	-0.034	0.056
**Nodule number†**	**0.672**	**0.605**	**0.739**	**1.067**	**0.832**	**1.302**	**0.395**	**0.183**	**0.607**

*Natural log transformed.

†Square-root transformed.

For each statistic, LCL = lower 95% confidence limit and UCL = upper 95% confidence limit; Diff PDI = PDI difference between species. Variables that differed significantly between hosts are in bold face.

### Association between bacterial genotypes and phenotypes

The matrices of pairwise phenotypic differences in *A. strigosus* shoot dry weight and average nodule area were each significantly associated with pairwise genetic differences at the *NifD* locus; association with the ITS region was not significant for shoot dry weight and marginal for average nodule area. Traits measured on *L. bicolor* exhibited no significant associations (Table 4).

**Table 4:** Mantel tests of the correlation between genetic distance matrices and phenotypic difference matrices

	***r***	***P***
**(a) ***A. strigosus* dry weight		
*NifD*	0.3190	**
*ITS*	0.0659	ns
**(b) ***A. strigosus* average nodule area		
*NifD*	0.413	***
*ITS*	0.128	*
**(c) ***L. bicolor* dry weight		
*NifD*	-0.006	ns
*ITS*	-0.038	ns
**(d) ***L. bicolor* average nodule area		
*NifD*	-0.0996	ns
*ITS*	-0.0280	ns

Significance, estimated from 50,000 permutations: ns*,* not significant*;* *, *p* < 0.05; **, *p* < 0.01; ***, *p* < 0.001.

### Fitness feedback between host and symbiont

For *A. strigosus*, there was an overall positive relationship between total nodule area and host dry weight (genotype mean correlation between total nodule area and host dry weight = 0.77, prob(H_0_: r = 0) < 0.0001). *Bradyrhizobium* genotypes that were more beneficial for *A. strigosus* also produced on that host fewer nodules (genotype mean correlation between nodule number and host dry weight = -0.35, p < 0.0001) that were larger (genotype mean correlation between average nodule area and host dry weight = 0.71, p < 0.0001).

A weak positive relationship between total nodule area and host dry weight (genotype mean correlation between natural log of nodule area and host dry weight = 0.36, prob(H_0_: r = 0) < 0.05) suggests a weaker but still positive fitness feedback between *L. bicolor* and *Bradyrhizobium* symbionts. In *L. bicolor*, there was no relationship between host dry weight and nodule number (genotype mean correlation between natural log of nodule number and host dry weight = 0.15, prob(H_0_: r = 0) > 0.4).

### Missing samples and cross-contamination

Nodule data were lost from 14 *L. bicolor* plants. Seven out of 100 control plants had nodules, indicating cross-contamination (perhaps water draining from containers was splashed from the greenhouse bench). These plants (two *A. strigosus* and five *L. bicolor*) were removed from analyses. Cross-contamination probably occurred late in the experiment because the nodulated control plants were still very small and had formed only a few small nodules. We are confident that these potential cross contaminations do not undermine our conclusions, since any broader cross contamination would only reduce phenotypic differences observed between *Bradyrhizobium* genotypes.

## Discussion

### Association richness and phylogenetic niche breadth

When growing wild, all four legume species that we examined specialized on subsets of the nodulating community of *Bradyrhizobium* genotypes. However, accounting for genetic breadth of symbionts in wild-collected nodules shifted the ranking of host specialization. When *Bradyrhizobium* genotype was ignored (i.e., by simply counting the total proportion of symbionts that were non-identical at the focal loci), *L. bicolor* ranked as most specialized and *L. arboreus* as most generalized. However, *Bradyrhizobium* isolates from wild-collected nodules were genetically clustered by host genus and each *Lupinus* species hosted a phylogenetically broader group of symbionts than did either *Acmispon* species. Clustering was strongest when isolates were categorized by *NifD* genotypes, which suggests that hosts respond to trait loci located on the *Bradyrhizobium* symbiosis island. The observation that species in *Acmispon* exhibit greater realized association specificity than do those in *Lupinus* agrees with previous surveys of wild-collected nodules from plants in these genera [66,78-80]. However, neither *L. bicolor* nor *A. strigosus* exhibited fundamental association specificity in greenhouse inoculation tests: both hosts could nodulate all tested *Bradyrhizobium* strains.

A gap between fundamental and realized association frequency is not uncommon [6] and indicates that environmental context strongly influences nodulation. The complex biotic community within natural soil could influence a genotype’s nodulation success [81-83]. For example, soil pathogens could trigger systemic acquired resistance in the host, which might affect nodulation by some rhizobial genotypes but not others [84].

The availability in soil of multiple rhizobial genotypes also provides scope for plants to actively prefer certain genotypes. Host-symbiont recognition signaling is a well-studied feature of legume-rhizobium interactions [85]. In particular, it has been hypothesized that, during nodule formation and prior to nitrogen fixation, plants can recognize and prefer more-beneficial genotypes via pre-infection partner choice; reviewed in [62]. It is unclear however, whether this host ability would be evolutionarily durable, as it requires genetic linkage between at least three sets of genes in two different organisms (nitrogen fixation genes and signaling genes in the bacterial genome and receptors to the bacterial signals in the host plant genome). Such linkage could be stable under very specific conditions, but could also be broken by any incident of recombination [86]. In several legume-rhizobium systems, including *A. strigosus*[87], multiple-genotype inoculation experiments have found that more-beneficial and/or co-evolved genotypes are more likely to occupy nodules [81,88,89]. However, none of these studies could definitively distinguish plant choice from interactions among rhizobia, which could be due to varying abilities to compete for soil resources [90], withstand direct interference by other genotypes [91], colonize the rhizosphere [92], and utilize a range of rhizosphere resources [93,94]. In experiments with *Bradyrhizobium* strains collected from our site, neither in vitro growth rate nor interstrain interference was correlated with nodulation rate on *A. strigosus*[87], but these and other hypotheses should be further tested in more complex conditions where bacterial traits not directly related to symbiont effectiveness might influence nodulation ability.

Realized association frequency might also be restricted by patchily distributed plant and symbiont genotypes [95-97], which could be caused by coevolution [98] and/or soil heterogeneity. In the Bodega Dunes, two episodes of Holocene dune advance [99,100] left contrasting soils that might structure both plant and *Bradyrhizobium* populations: *L. bicolor* is restricted to mid-Holocene dunes whereas *A. strigosus* and *A. heermannii* occur only on poorly stabilized late-Holocene dunes (E. L. S. and T. J. M., personal observation). In contrast, *L. arboreus* occurs across dunes of both ages [101]. We specifically sampled nodules from *L. arboreus* across its habitat range to control for potential confounding of host and *Bradyrhizobium* distribution; indeed, *L. arboreus* nodules harbored genotypes from across each gene network. However, the genotypic composition of isolates from *L. bicolor* did not differ significantly from those isolated from *L. arboreus*, which suggests that soil habitat is not the only determinant of genotype in wild-collected nodules of *Lupinus*.

### Response and effect specificity, fitness trade-offs, and fitness feedback

The “Jack-of-all-trades is a master of none” hypothesis was supported by evidence that specialist *A. strigosus* obtained more fitness benefit from its genetically narrower group of beneficial symbionts than generalist *L. bicolor* obtained from its genetically broader group of compatible symbionts.

As predicted by the co-adaptation hypothesis, each host received greater fitness benefit from genotypes isolated from congeners or conspecifics (genotypes isolated from *L. bicolor* provided particularly poor benefit to *A. strigosus*). Although the sample sizes used to estimate fitness are not large, this pattern of response specificity has been detected in several studies of unmanaged legumes [102-104] and suggests that symbiont effectiveness may be increased by host-symbiont coevolution [45,89,98,105].

*A. strigosus* plants were highly specialized in their fitness effect on the tested *Bradyrhizobium* genotypes, which is consistent with previous evidence that this host imposes absolute sanctions [64] on less effective genotypes [87]. There was much less variance among *Bradyrhizobium* genotypes in the benefits they obtained from *L. bicolor*.

Also supporting the co-adaptation hypothesis, genotypes of *Bradyrhizobium* were better adapted to hosts related to those from which their ancestors were isolated. This pattern was strongest on *A. strigosus*: genotypes isolated from congeneric hosts produced greater total nodule area and therefore likely yielded more progeny than did genotypes isolated from lupines.

As a consequence, symbiont benefits to *A. strigosus* fitness positively fed back to symbiont fitness via increased allocation to nodules: genotypes beneficial to this host realized greater fitness benefits than did those that were not. An overall positive correlation between *NifD* genotypic mean values of total nodule area and host shoot dry weight on *A. strigosus* suggests that strong positive fitness feedback between mutualists in this partnership [67] is driven by traits encoded in the symbiosis island. This feedback pattern was much weaker on the generalist, *L. bicolor*, and was not significantly correlated with *ITS* genotype.

Positive partner feedback can produce positive frequency dependence and drive the most beneficial partners to local fixation [49,106], a process that has been predicted for other legumes that exhibit symbiont specificity [83].

Much stronger positive fitness feedback might allow specialist *A. strigosus* to outcompete generalist *L. bicolor* in local patches [107]. However, if *L. bicolor* were a better colonizer, which is expected of generalists [108], then it might persist in a spatially patchy environment through a competition-colonization trade-off [109]. In that case, *A. strigosus* and *L. bicolor* would not coexist at a local scale, but both might persist at a larger spatial scale via patch dynamics. This process could also spatially structure *Bradyrhizobium* populations [12]. Indeed, wild *A. strigosus* plants were found in association with a genetically narrow range of *Bradyrhizobium* genotypes that were most beneficial to their growth, suggesting that these genotypes dominate *Bradyrhizobium* populations in areas inhabited by this host. In contrast, as predicted from the weaker correlations between plant and rhizobial fitness components on *L. bicolor*, wild *L. bicolor* plants associated with a genetically wider range of *Bradyrhizobium* genotypes that were not necessarily the most beneficial.

## Conclusions

A field survey detected differences in partner specificity among four sampled host species and greenhouse experiments revealed that at this site *A. strigosus* clearly specializes on a genetically narrower range of symbionts than does *L. bicolor*. The fitness benefits that these two hosts received from symbiotic partners affirmed two important tenets of evolutionary theory. First, as predicted by the Jack-of-all-trades is a master of none hypothesis, specialist *A. strigosus* obtained greater benefit from its genetically narrow group of beneficial symbionts than generalist *L. bicolor* obtained from a genetically more diverse group. Second, as predicted by the co-adaptation hypothesis [2,3,50], each test species performed better with partner genotypes isolated from conspecifics. Further, host fitness benefit translated directly into symbiont fitness via increased allocation to nodules: nodules occupied by genotypes isolated from conspecific hosts received the greatest benefit. These patterns were strongest in *A. strigosus*. Positive fitness feedback between this specialized host and its co-adapted symbionts could drive positive plant-soil feedback and contribute to natural distribution patterns observed in the field.

## Methods

### *Collection of wild* Bradyrhizobium *isolates*

During the end of March and the beginning of April 2007, we isolated *Bradyrhizobium* bacteria from nodules of four native California legumes growing on the Bodega Dunes at Bodega Marine Reserve (38°19′01″N, 123°04′18″W) and Sonoma Coast State Park (38°20′34″N, 123°03′32″W). Portions of these dunes were stabilized in the mid-twentieth century by planting European beach grass, *Ammophila arenaria*[110,111], but no samples were collected in areas occupied by *A. arenaria*. The four host legumes belong to two genera, *Acmispon* (*A. heermannii* Greene [112], and *A. strigosus* Nutt. Ex Torr. & Gray [112], previously *Lotus heermannii* and *Lo. strigosus*, respectively) and *Lupinus* (*L. bicolor and L. arboreus*), which are from distantly related clades of papilionoid legumes [113].

We harvested and cultured isolates from up to 20 nodules from each of four to six seedlings of each host species using the procedures of Sachs and colleagues [66]. Briefly, intact plants were excavated, the root systems washed in tap water; nodules excised, surface sterilized, rinsed, crushed and individually streaked onto two replica plates of solid modified arabinose-gluconate medium (MAG). Successful cultures were archived in 25% glycerol-MAG at -80°C.

### Molecular methods and analysis

Genomic DNA was purified from 25 μl of each frozen isolate using Gentra Puregene Yeast/Bacteria kits, (Qiagen, Valencia, CA). We PCR amplified the intragenic spacer between 16 s and 23 s ribosomal subunits (*ITS*, 1256 nt) [114] and portions of the nitrogenase α-subunit gene (*NifD*, 756 nt) [115] as previously described [66]. Amplification products were sequenced in both directions using an Applied Biosystems 96 capillary 3730xl DNA Analyzer (Foster City, CA) at the University of California, Berkeley, Sequencing Facility. Two to eight isolates from each plant were successfully amplified and sequenced, which ultimately yielded a sample of 84 sequenced isolates (Additional file 1: Table S1).

Sequences were aligned using MAFFT [116] with default parameters. Genotypes were identified in MacClade 4.05 [117]. Some portions of the *ITS* included indels and these regions were removed from the analysis.

### *Quantifying realized* in situ *association specificity*

Specialization is most easily quantified as the number of taxa with which a taxon interacts [118], termed “partner richness”. However, this measure accounts for neither association frequency [72,73], phylogenetic distance among partners, e.g., phylogenetic breadth [28], nor fitness effects of interactions. Resurging interest has stimulated new methods for quantifying ecological specialization [1,71,75]; we used the Paired Differences Index.

From field collected samples, we estimated the *in situ* link strength of each pairwise interaction, *P*_*ij*_, as the proportion of nodules sampled from the *i*^th^ host species occupied by the *j*^th^*Bradyrhizobium NifD* type:

(1) $$P_{i,j}=\frac{n_{i,j}}{n_{i}}$$

where *n*_*i,j*_ = the number of nodules on the *i*^th^ host species occupied by the *j*^th^*Bradyrhizobium NifD* type and *n*_*i*_ = the total number of nodules sampled from the *i*^th^ host (*n*_*ACHE*_ = 19, *n*_*ACST*_ = 18, *n*_*LUAR*_ = 21, *n*_*LUBI*_ = 23).

Observed specificity of each of the four hosts was then measured as the differential frequency of interaction with the 22 different available *NifD* types, using the Paired Differences Index (PDI) measure [71]:

(2) $$\mathrm{PD}I_{i}=\frac{\sum_{j=2}^{N}\left(P_{i,1}-P_{i,j}\right)}{n_{i}-1},$$

where *P*_*i*,1_ is the strength of the strongest link with the *i*^th^ host. The PDI values were not normally distributed; therefore, to compare PDIs of the four hosts, we estimated confidence limits from 1000 simulated 20-nodule populations generated as follows. Using the RAND (“TABLE”,) function of SAS® 9.3, we specified the frequency with which each genotype appeared in each simulated population, assuming that hosts randomly sampled rhizobia and therefore the observed frequencies of the 22 *NifD* genotypes in the total sample of 81 nodules represented their availability in the *Bradyrhizobium* population. From each simulated population, we calculated the joint PDI for all four hosts together:

(3) $$\mathrm{PD}I_{\text{joint}}=\frac{\sum_{j=2}^{N}\left(P_{1}-P_{j}\right)}{N-1},$$

where *P*_*j*_ is the link strength averaged across the four hosts of *Bradyrhizobium* strain *j*, *P*_1_ is the average link strength of the *Bradyrhizobium* strain with the strongest link averaged over the four host species, and *N* = 20 nodules, then used these values to estimate the mean and variance of PDI and calculate 95% confidence limits.

### Genetic breadth of partners isolated from wild-grown hosts

We constructed a molecular network for each DNA region with the neighbor-net algorithm implemented in SplitsTree 4.8 [119], using the Akaike Information Criterion (AIC) implemented in Modeltest (version 3.4) to choose a suitable model of sequence evolution [120]. The *NifD* genotype network was estimated using the GTR + I model [121] of sequence evolution and the *ITS* network was estimated with the HKY + I + G model [122]. When there were multiple isolates with the same sequence, we randomly picked one for analysis.

A molecular network approach was favored over a phylogenetic tree approach, as previous studies have repeatedly found incongruence between phylogenies of different gene loci in *Bradyrhizobium* bacterial strains [66,79,123-125]. Moreover, in our sample, SplitsTree found significant recombination in the *ITS* region and marginally significant recombination within the *NifD* locus. Genetic networks are standard tools for the analysis of populations in which genomes recombine; such events will induce a reticulated representation of genealogies, which violates the bifurcating model of phylogenetic trees, as reviewed in [126].

For each DNA region, we computed the matrix of average number of differences between pairs of sequences (pairwise differences) within and between host genera to test whether bacterial communities harbored by the two genera differed in genetic diversity. We further computed matrices of within and between host-species pairwise Φ_st_[127] to test whether pairs of host species harbored genetically distinct groups of bacteria. The Φ_st_ statistic for nucleotide diversity is analogous to Wright’s F_ST_, but accounts for sequence divergence as well as genotype frequency. Finally, each DNA region was analyzed with a two-level hierarchical AMOVA model with the following levels: within host species, between species within host genus, and between host genera. Significance tests for each level of the AMOVA, the Φ_st_ values, and the pairwise differences were obtained by permuting genotypes between levels or populations. These analyses were performed with Arlequin 3.0b [128].

### Greenhouse experiment

#### Seed source

Seeds of *A. strigosus* and *L. bicolor* were obtained from ripe fruits of many plants growing at Bodega Marine Reserve in May-June 2008, pooled, mixed and stored dry at room temperature. In mid-July, seeds were surface-sterilized and scarified [66,129], then germinated individually in wells of 96-well culture plates containing 200 ml of sterile ddH_2_O (incubated at 15°C, 5–7 days). Seedlings of *A. strigosus* were transferred to bleach-sterilized 38-mm × 140-mm Conetainers (Stuewe & Sons, Corvallis, OR, USA) filled with autoclaved quartzite sand [66]. One-week old seedlings of *L. bicolor* were transferred to bleach-sterilized 64-mm × 250-mm Deepots (Stuewe & Sons, Corvallis, OR, USA) filled with autoclaved calcine-clay (Turface MVP®, Profile Products, Buffalo Grove, IL, USA). Media were chosen to maximize plant survival. Neither medium provides nitrogen; we have no reason to expect that differences in media would affect symbiont specificity or fitness benefits to hosts.

All transplants were placed in a greenhouse under ca. 50% shade for seven days of hardening, with misting twice daily, and subsequently exposed to full sunlight, watered daily, and fertilized weekly with 10-ml Jensens’s nitrogen-free solution [130] until treatments were applied one month after germination (see below).

#### Preparation of bacterial inocula

For the greenhouse inoculation experiment, we chose 29 *Bradyrhizobium* strains that represented the range of a preliminary concatenated *nifD* and *ITS* network of the sequenced isolates. For reference to previously described *Bradyrhizobium* genotypes, we added two strains (T11 and T12) from among those described by Sachs and colleagues [66], for a total of 31 strains. A culture of each strain was initiated from ~50 μl of original stock archived in glycerol, plated onto solid MAG medium [78] and incubated at room temperature until bacteria covered the plate. Bacteria were washed from plates into 50-ml polypropylene tubes containing sterile dd H_2_O and vortexed. Bacterial concentration of each inoculum was estimated via light absorbance at 600 nm with a spectrophotometer and a previously established standard growth curve; then adjusted to 10^8^ cells ml^-1^ by dilution in sterile dd H_2_O. Each treatment plant received 10 ml of bacterial suspension and control plants received 10 ml of sterile dd H_2_O.

### Experimental design

The two host species were planted and inoculated at the same time, but grown in different substrates and separated on the greenhouse bench to prevent shading of *A. strigosus* by the larger *L. nanus*. Each host species was arranged into ten 36-seedling blocks: 31 test seedlings, each randomly assigned to a *Bradyrhizobium* strain, and five seedlings that received the sterile water control inoculum. All treatments were spatially randomized within each block and the 10 blocks were randomly located on the greenhouse bench, for a total of 360 plants per host species. Seven to eight weeks after inoculation, plants were harvested to obtain oven-dried aboveground (shoot) dry weight (a component of plant fitness). We counted, excised, and photographed nodules with a stage micrometer to obtain their total projected area using ImageJ64 [131]. Because symbiotic rhizobia reproduce clonally and are ultimately released from legume nodules into the soil, nodule number and mass or projected area have been treated as multiplicative components of rhizobial fitness [67,129,132-134]. When obtained using a standard-density, single-genotype inoculum, nodule number indexes the probability that an individual rhizobium cell can initiate a nodule [88,135]. Nodule size, measured as biomass or projected area, is a good proxy for the number of viable rhizobia in a nodule [129,132,136]. Since most nodules are initiated by a single rhizobium cell [88,137], nodule size indexes rhizobium fecundity. Like many fitness components, nodule number and individual nodule size are often negatively correlated [138]. Nonetheless, when all nodules on a host are occupied by a single genotype, the ratio of total nodule area to nodule number estimates average fecundity on that host for cells of that genotype.

### Analysis

#### Fundamental association specificity

No analysis was required because all hosts nodulated all *Bradyrhizobium* genotypes.

#### Host response specificity

Because the experimental design technically precludes treating test host as an independent variable in the ANOVA, it was most conservative to analyze each host species separately. These models (Additional file 2: Table S2) included random effects of spatial block and *Bradyrhizobium* genotype, with genotype nested within host species from which it was isolated and further nested within host genus of isolation, along with all appropriate interactions. A planned one degree-of-freedom contrast compared the growth of inoculated versus uninoculated test plants and a posterior 1-df contrast compared the growth of plants inoculated with bacterial genotypes that had been isolated from the each of the two plant genera (*i.e*., *Lupinus*, *L*, versus *Acmispon*, *A*). Bacterial genotype effects on dry weight (Additional file 3: Table S3) were compared using a Tukey-Kramer test of honestly significant pairwise differences [139].

Although the experimental design technically precludes treating test host as an independent variable in the ANOVA, if the different growth conditions (different growth media and locations in the greenhouse) of the two test host species did not interact with the effect of *Bradyrhizobium* genotype, then both species might be analyzed with a single model. Lack of replication of *Bradyrhizobium* genotype within blocks precluded testing for this interaction within each species. Nonetheless, we cautiously tried this less conservative approach, in which a significant interaction between test host species and *Bradyrhizobium* genotype G × G interaction [77], would indicate differences in response specificity between *L. bicolor* and *A. strigosus*.

We analyzed the entire data set (both host species) by adding the fixed factor of test host and all its interactions to a nested hierarchy of generalized linear mixed models to examine how host species, *Bradyrhizobium* genotypes, the species or genus of plant from which they were isolated, and two-way interactions affected plant benefit. The two hosts differed so much in response specificity that they exhibited very different distributions of shoot weight. For this reason, all models employed a gamma probability density function and a logit link function. Corrected Akaike Information Criteria (AICc) were used to find the best model (i.e. the one with the lowest AICc). Relative likelihoods of other models were compared using e^(AICmin-AIC*i*)/2^, where AIC_min_ is the corrected AICc of the best-fitting model and AIC_i_ is the AICc of the model being compared [140]. This analysis was performed with SAS Version 9.3 (SAS Institute Inc., 2002–2010, Cary, NC, USA). The best fitting model (model 16 in Additional file 4: Table S4) included the fixed effects of host species (host), host species from which the *Bradyrhizobium* genotype was isolated (sp_orig), and the host species by species of origin interaction, random effects of block (block) and *Bradyrhizobium* genotype nested within the host species from which the genotype was isolated (strain(ge_orig)), and the interaction of host species with *Bradyrhizobium* genotype nested within host species of origin (host* strain(sp_orig)). Thus, the G × G interaction was split between a fixed effect interaction (Additional file 5: Table S5) and a random effect interaction (Additional file 7: Table S7).

We also compared test host growth response, relative to uninoculated control, to *Bradyrhizobium* genotypes isolated from conspecifics versus response to those isolated from allospecifics. To calculate growth response (fold increase over uninoculated control), we used shoot weight of the control plants in each block to calculate the mean growth effect on each test host of the *i*^th^ genotype in each block: (*w*_*i*_ – *w*_0_)/*w*_0_, where *w*_*i*_ = shoot dry weight of the plant inoculated with genotype *i* and *w*_0_ = shoot dry weight of the uninoculated conspecific control plant. This variable was analyzed with a generalized linear model including test host, host species of origin, and their interaction, with an exponential distribution and canonical (reciprocal) link function. A planned contrast was used to compare the fitness benefit obtained from *Bradyrhizobium* genotypes isolated from conspecific versus heterospecific hosts. This analysis was performed with JMP Version 10.0.0 (SAS Institute Inc, Cary, NC, 1989–2007).

To quantify specialization using the host fitness responses, we calculated the fitness effect of each *Bradyrhizobium* genotype on each test host species separately for each of the *k* = 10 blocks,

(4) $$p_{\mathrm{ijk}}=\frac{w_{\mathrm{ijk}}-w_{\mathrm{iCk}}}{w_{\mathrm{ijk}}}$$

where *w*_*ijk*_ = shoot dry weight of the *i*th host species inoculated with the *j*th strain in the *k*th block, and *w*_*iCk*_ = shoot dry weight of the uninoculated conspecific control plant in the same block. The Paired Differences Index (PDI) [71] of the *i*th test host can then be calculated for each block by ranking within-block values of *p*_*ijk*_ from highest (*j* = 1) to lowest (*j* = *N*), where *N* = 30 tested strains, to obtain:

(5) $$\mathrm{PD}I_{\mathrm{ik}}=\frac{\sum_{i=2}^{N}\left(p_{i,1,k}-p_{\mathrm{ijk}}\right)}{N-1},$$

Variance among blocks was used to calculate 95% confidence intervals for the PDI of each test host species.

#### Specificity of host effect on symbionts

The effect of the two test hosts on the fitness of each bacterial genotype was examined by univariate ANOVAs on nodule number, total nodule area per plant, and average nodule size. All dependent variables were log transformed to meet the ANOVA normality assumptions [141], pp. 185, 202–204. Standard deviations were not equal among factor levels; so we performed a Welch ANOVA, which allows for heteroscedasticity among factors [141], pg. 183.

Specificities of effect of the two test hosts on *Bradyrhizobium* genotype fitness under conditions of individual inoculation were calculated from equation (4), above, by defining *p*_*ijk*_ in terms of each of two rhizobial fitness components, nodule number and total projected area of nodules. To meet the assumptions of normality and homoscedasticity between populations, nodule number was natural log transformed and nodule area was square root transformed. Variance among blocks was used to calculate 95% confidence intervals.

#### Test of association between bacterial genotypes and phenotypes

The groups of *Bradyrhizobium* genotypes isolated from congeneric versus heterogeneric hosts were identified by the population genetic analysis. The *A* group (genotypes T1 - T12) were originally isolated from *Acmispon*; the *L* group (genotypes T13 - T31) were originally isolated from *Lupinus*. Having been classified by host association, the bacteria within one group may not be more related to each other than to bacteria in the other group. Thus, although this contrast compares the average effects of the two bacterial groups, it does not test for a specific association between bacterial genotype and phenotype. To explicitly test for an association between bacterial genotype and symbiotic phenotypes, we used Mantel tests of non-random association between matrices. We computed six matrices: two matrices of pairwise genetic distances between all pairs of genotypes, one for *NifD* and one for *ITS*, and four matrices of pairwise mean phenotypic differences between all pairs of bacterial genotypes, two for *A. strigosus* and two for *L. bicolor.* Each genetic distance matrix was then compared with the four different phenotype matrices. Under the null hypothesis of these tests, a random association between the elements of the phenotype matrix and those of the genotype matrix would indicate that across *Bradyrhizobium* genotype pairs, the two genotypes within a pair shared phenotypes independently of the genetic distance between them. In contrast, a positive, non-random association would indicate that the phenotypic difference between the genotypes in a pair increases in concert with an increase in their genetic distance. The two reference genotypes, T11 and T12, were omitted during computation of the matrices, as was genotype T30, which we were unable to sequence at the *NifD* locus. The ANOVAs were performed with JMP®, Version 7. (SAS Institute Inc., Cary NC, 1989 – 2007); the Mantel tests were performed with the R statistical software [142].

#### Fitness feedback between host and symbiont

To estimate fitness feedback between host and symbiont, the JMP statistical package was used to calculate the Pearson product mean correlation between host fitness, measured as shoot dry mass, and symbiont fitness, measured as nodule number, total nodule area per plant, and average nodule size.

### Availability of supporting data

The sequence datasets supporting the results of this article are available from Genbank (http://www.ncbi.nlm.nih.gov/genbank/); accession numbers JQ230720 – JQ230882.

## Abbreviations

χ2: Chi-square test statistic; A: *Acmispon*; ACHE: *Acmispon heermannii*; ACST: *Acmispon strigosus*; AIC: Akaike information criterion; AMOVA: Analysis of molecular variance; ANOVA: Analysis of variance; d.f.: degrees of freedom; dd H2O: double-distilled water; DNA: Deoxyribonucleic acid; F: F-test statistic; G × G: Genotype by genotype interaction; GTR + I: Generalized time-reversible model of DNA evolution with invariant sites (I); HKY + I + G: Hasegawa, Kishino and Yano model of DNA evolution with invariant sites (I) and Gamma-distributed among site rate variation (G); ITS: 16S-23S rRNA internal transcribed spacer; L: *Lupinus*; LUAR: *Lupinus arboreus*; LUBI: *Lupinus bicolor*; MAG: Modified arabinose gluconate; nt: nucleotides; NifD: Gene coding for the alpha subunit of dinitrogenase; p: probability of obtaining a test statistic at least as extreme as the one observed, assuming a true null hypothesis; PDI: Paired differences index; Φst: The correlation of haplotypic diversity at different levels of hierarchical subdivision.

## Competing interests

The authors declare that they have no competing interests.

## Authors’ contributions

With advice from ELS and assistance from JLS and TJM, ME collected, isolated, archived and genotyped all but two of the samples (which had been collected by JLS), conducted the experiments and analyzed the genetic data. ELS and SSP calculated the paired difference indices, and ELS and ME analyzed the phenotypic data. ME wrote the manuscript with advice and assistance from ELS, who subsequently extensively revised the final manuscript. All authors read and approved the final manuscript.

## Supplementary Material

Additional file 1: Table S1 *Title of data:* Field-sampled nodules, their source host, identifying symbols, and accession numbers for *NifD* and *ITS* sequences submitted to GenBank. *Description of data:* * host = host species, pln = plant within species, nod = nodule on plant, strain = strain identifier, T-type = inoculation type in greenhouse experiment (blank cells indicate strains not used in that experiment), g’type *NifD* and g’type *ITS* identify the *NifD* and *ITS* genotypes, respectively, included on Figure 1. Respective *NifD* and *ITS* GenBank sequence IDs and accession number are indicated as seq *nifD*, seq *ITS*, accession # *NifD*, and accession # *ITS*. Blank cells in these columns indicate isolates that did not amplify. Click here for file

Additional file 2: Table S2 *Title of data:* Separate ANOVA for each host species of plant and bacterial fitness-components. *Description of data:* Significance: ns, not significant; **, *p*<0.01; ***, *p*<0.001. ^1^ Degrees of freedom for F-test of random block effect: numerator = 9; denominator for (a) = 271, for (b) = 256. ^2^ Degrees of freedom for F-test of random genotype effect: numerator = 30, denominator for (a) = 271, for (b) = 256. ^3^ Tests of contrast between *Bradyrhizobium* groups *A* (isolated from *Acmispon*) and *L* (isolated from *Lupinus*); degrees of freedom: numerator = 1, denominator for (a) = 271, for (b) = 256. Click here for file

Additional file 3: Table S3 *Title of data:* Tukey-Kramer honestly significant differences in trait values between *Bradyrhizobium* genotypes inoculated on two host species, (a) *A. strigosus*, (b) *L. bicolor. Description of data:* Genotypes sharing the same capital letter are not statistically significantly different at p < 0.05. * shoot dry weight. Click here for file

Additional file 4: Table S4 *Title of data:* Generalized linear mixed models fitted to variance in host shoot dry weight. *Description of data:*^+^ estimated G-matrix not positive definite. Host = test host species, block = spatial block, strain = *Bradyrhizobium* genotype, sp_orig = host species from which *Bradyrhizobium* genotypes were isolated, ge_orig = host genus from which *Bradyrhizobium* genotypes were isolated. All models assumed a gamma distribution with a logit link function. Model 16 (bold face) provided the best fit (i.e., minimized AICc). Adding the block by host species interaction improved the fit (e^(AICmin-AICi)/2^ = 5.21 x 10–13) but the proportion of variance explained by this component (4.91% + 2.42%, Additional file 7: Table S7) was only marginally significant. The next best fitting model (model 1) included the fixed effect of host and the random effects of block and *Bradyrhizobium* genotype, with interactions of host with strain and with block. Residuals plots suggested that this model did not perform much worse than the best model, but nesting *Bradyrhizobium* genotype into host species from which it was isolated greatly improved the relative likelihood that the model fit the data (model 16 versus model 1; e^(AICmin-AICi)/2^ = 1.28 × 10–12)). Examination of Q-Q plots revealed that all other models provided much poorer fits to the data. Click here for file

Additional file 5: Table S5 *Title of data:* ANOVA table of Type III tests of fixed effects on test host shoot dry weight. *Description of data:* From model 16 of Additional file 4: Table S4. Click here for file

Additional file 6: Table S6 *Title of data:* Differences of least square means (logits) of shoot dry weight of test hosts by host of origin. *Description of data:* From model 16 in Additional file 4: Table S4. Test host species: ACST = *A.strigosus*; LUBI = *L. bicolor*. Origin host species: ache = *A. heermannii*; acst = *A.strigosus*; luar = *L. arboreus*; lubi = *L. bicolor*. Click here for file

Additional file 7: Table S7 *Title of data:* Covariance parameter estimates for random factors associated with test host shoot dry weight. *Description of data:* From model 16 of Additional file 4: Table S4. Click here for file

Additional file 8: Table S8 *Title of data:* Least squares means (logits) of shoot dry weight of test plants inoculated with genotypes isolated from the four hosts. *Description of data:* From model 16 of Table 3. Click here for file
